# Supplementary material for: USP7 Induces Chemoresistance in Triple-Negative Breast Cancer via Deubiquitination and Stabilization of ABCB1
Source: Cells. 2022 Oct 19;11(20):3294. doi: 10.3390/cells11203294 (PMC9600340; doi:10.3390/cells11203294)
Supplement: Supplementary file 1 [file cells-11-03294-s001.zip › cells-1881114-supplementary.pdf]

Supplementary Figures

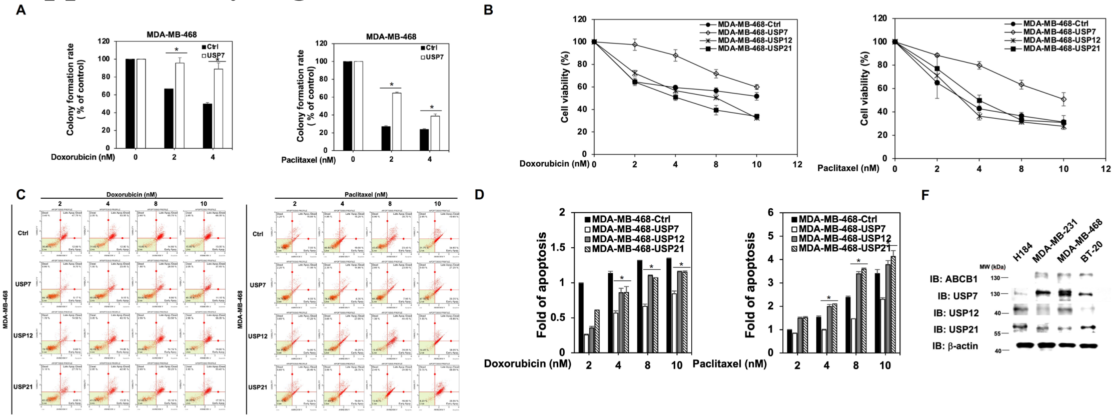

Supplementary figure S1. The role of USP family members in MDA-MB-468 cells under chemo-drug treatment. (A) Analysis of colony formation in MDA-MB-468 cells with transient expression of USP7 under doxorubicin and paclitaxel treatment, respectively. Analysis of cell viability (B) and apoptotic (C, D) assays in MDA-MB-468 cells with transient expression of USP7, USP12, and USP21 under doxorubicin (left) and paclitaxel (right) treatment, respectively (F) Expressions of ABCB1, USP7, USP12, and USP21 in breast cell lines. *: *P*-value <0.05, compared with control cells.

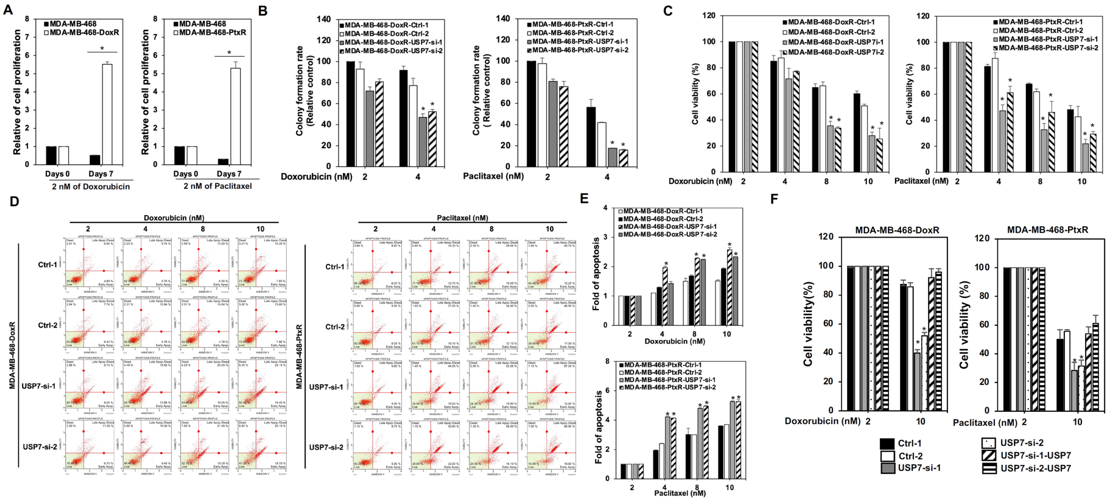

Supplementary figure S2. The role of USP7 in chemoresistant activity of MDA-MB-468 cells with chemo-drug resistance. (A) Analysis of the viability of doxorubicin-resistant MDA-MB-468 (left) and paclitaxel-resistant MDA-MB-468 (right) cells treated with 2 nM of doxorubicin or 2 nM of paclitaxel for 7 days compared with parental MDA-MB-468 cells, assessed using MTT assays. Analysis of colony formation (B), viability (C), and apoptotic (D, E) activities in doxorubicin- and paclitaxel-resistant MDA-MB-468-USP7-silencing cells under doxorubicin and paclitaxel treatment. (F) Cell viability of doxorubicin- and paclitaxel-resistant MDA-MB-468-USP7-silencing cells with re-expression of USP7 under 10 nM of doxorubicin and paclitaxel treatment, respectively. * *P*-value <0.05, compared with control cells.

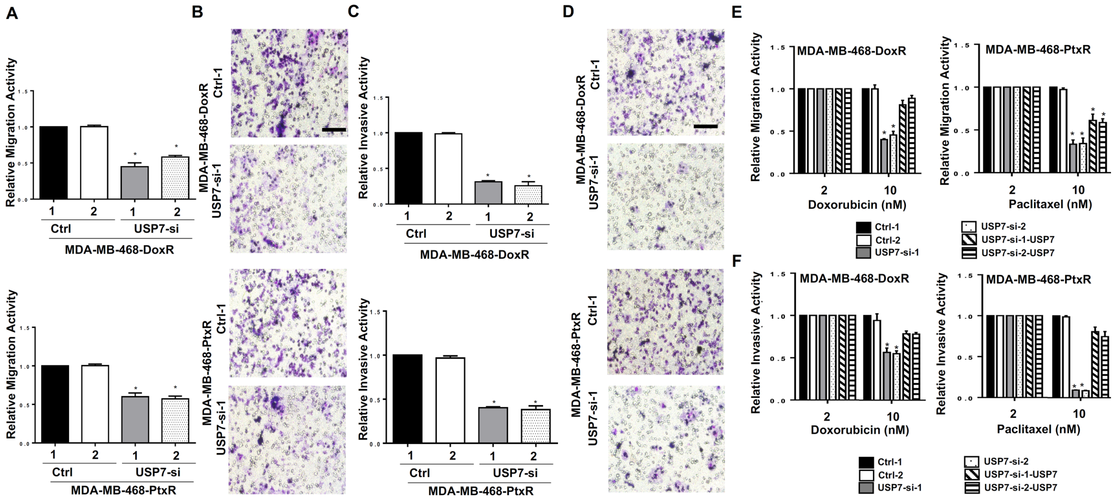

Supplementary figure S3. The role of USP7 in migration and invasive activity of chemo-drug-resistant MDA-MB-468 cells. Analysis of migration (A, B) and invasive (C, D) activities in doxorubicin- and paclitaxel-resistant MDA-MB-468-USP7-silencing cells. Analysis of migration (E) and invasive (F) activities in doxorubicin- and paclitaxel-resistant MDA-MB-468-USP7-silencing cells with re-expression of USP7. *: *P*-value <0.05, compared with control cells.

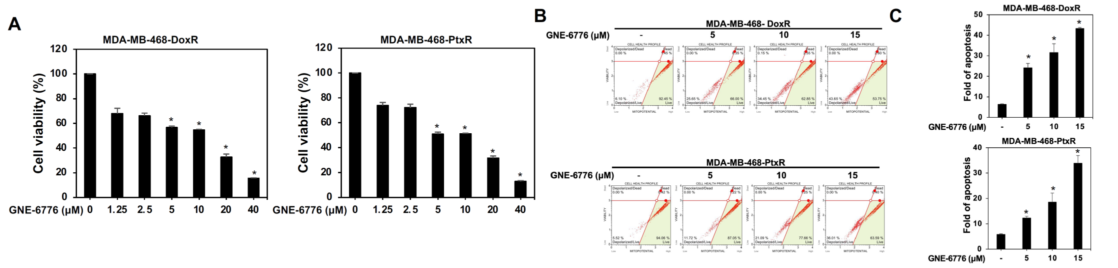

Supplementary figure S4. The effect of USP7 inhibitor on chemo-drug-resistant MDA-MB-468 cells. Analysis of viability (A), and apoptotic activities (B, C) in doxorubicin- and paclitaxel-resistant MDA-MB-468 cells under GNE-6776 treatments. *: *P*-value <0.05, compared with untreated cells.

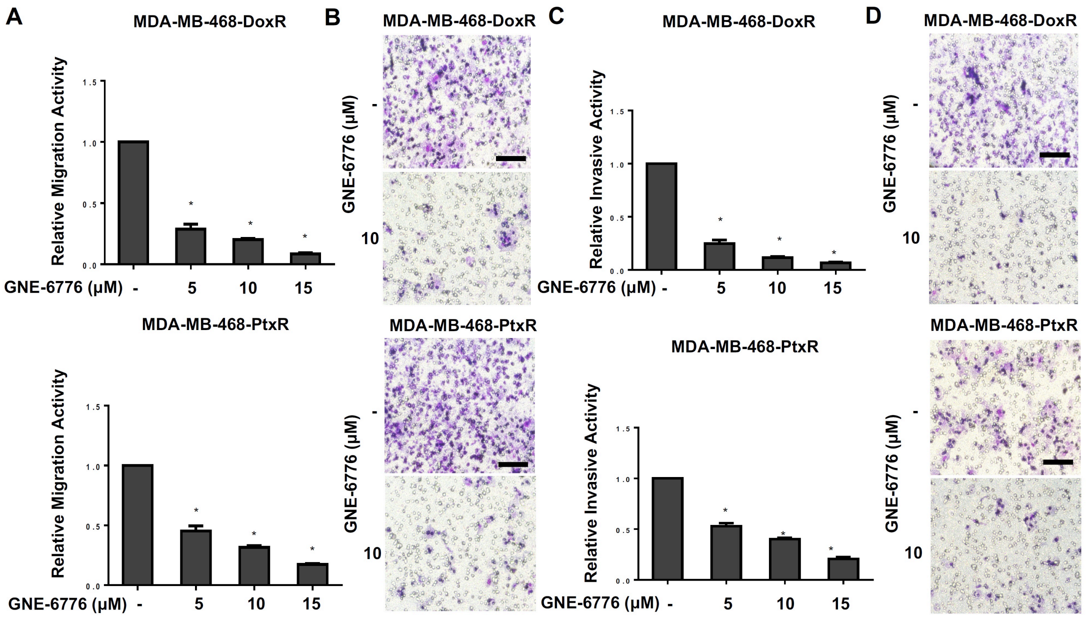

Supplementary figure S5. The effect of USP7 inhibitor on migration and invasive activities of chemo-drug-resistant MDA-MB-468 cells. Analysis of migration (A, B) and invasive (C, D) activities in doxorubicin- and paclitaxel-resistant MDA-MB-468 cells under GNE-6776 treatment. *: P-value <0.05,

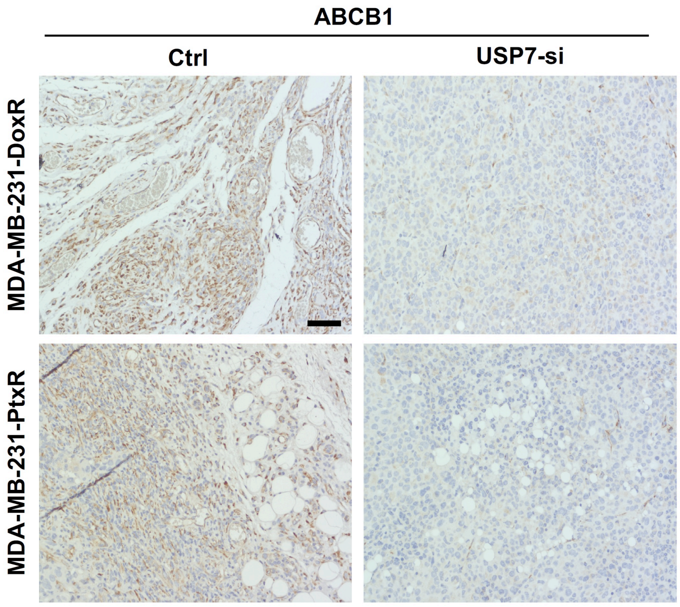

Supplementary figure S6. Correlation between USP7 and ABCB1 *in vivo*. Immunohistochemistry staining analysis for ABCB1 in the xenograft system injected with doxorubicin- and paclitaxel-resistant MDA-MB-231-USP7-silencing cells by using using anti- ABCB1 antibodies. Scale bar represented 200 μm.

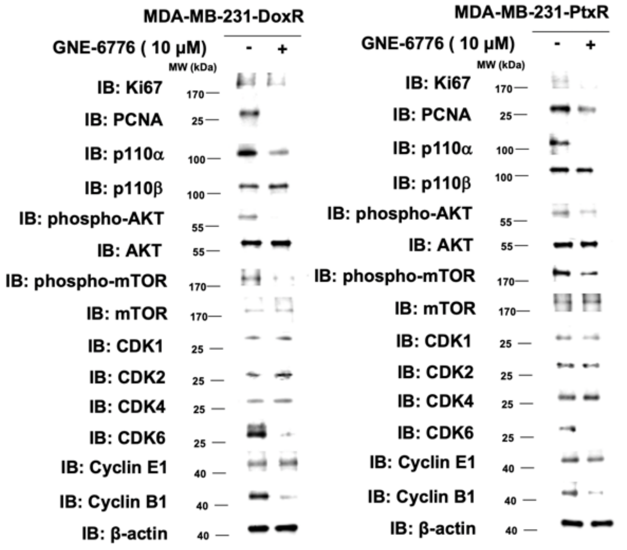

Supplementary figure S7. Expression of proliferation markers, PI3K/AKT/ mTOR signaling, and cell cycle markers in chemo-drug-resistant MDA-MB-468 cells. The levels of proliferation markers (Ki67 and PCNA), PI3K/AKT/mTOR signaling (p110a, p110b, phosphor-AKT. AKT, phosphor-mTOR, and mTOR), and cell cycles markers (CDK1, CDK2, CDK4, CDK6, Cyclin E1, and Cyclin B1) in doxorubicin- and paclitaxel-resistant MDA-MB-468 cells under 10 mM of GNE-6776 treatment.

Supplementary Method

Immunohistochemistry (IHC)

Four μm thick sections of all tissue were cut from the paraffin-embedded specimens for IHC staining. The samples were fixed in 4% paraformaldehyde solution for 24 h. the IHC was performed by by using Mouse and Rabbit Specific HRP/DAB IHC Detection Kit - Micro-polymer (Abcam, ab236466). Briefly, the endogenous peroxidase activity was eliminated with 3% hydrogen peroxide and then incubated with 1% bovine serum albumin and 5% normal goat serum for the blocking step. After reacting with a biotinylated secondary antibody for 1.5 h, antigen-antibody reactions were visualized using streptavidin-horseradish peroxidase conjugate with DAB chromogen. All slides were counterstained with hematoxylin. The antibodies used in IHC were listed in supplementary table 1.

Supplementary Tables

Supplementary Table S1. List of proteins tested by and characteristics of the corresponding antibodies

Protein	Assay	Origin	Dilution	Incubation period
E-cadherin	WB	#3195, Cell signaling	1:500	4℃, Overnight
Vimentin	WB	A19607, ABclonal	1:500	4℃, Overnight
N-cadherin	WB	#610920, BD biosciences	1:500	4℃, Overnight
Plakoglobin	WB	ab184919, abcam	1:2000	4℃, Overnight
β-actin	WB	#3700, Cell Signaling	1:2000	4℃, Overnight
Bcl-2	WB	#2872, Cell Signaling Technology	1:500	4℃, Overnight
Bcl-xL	WB	#2764, Cell Signaling Technology	1:500	4℃, Overnight
BAX	WB	#2772, Cell Signaling Technology	1:500	4℃, Overnight
BIM	WB	#2819, Cell Signaling Technology	1:500	4℃, Overnight
cleaved PARP	WB	#5625, Cell Signaling Technology	1:500	4℃, Overnight
cleaved Caspase-7	WB	#9491, Cell Signaling Technology	1:500	4℃, Overnight
cleaved Caspase-3	WB	#9661, Cell Signaling Technology	1:500	4℃, Overnight
cleaved Caspase-9	WB	#9505, Cell Signaling Technology	1:500	4℃, Overnight
Flag	WB,IP	TA50011-100,Origene	1:500	4℃, Overnight
HA	WB, IP	#05-904, millipore	1:500	4℃, Overnight
USP7	WB	A300-033A, Bethyl	1:2000	4℃, Overnight
Ub K48	WB	#05-1307, millipore	1:500	4℃, Overnight
t-Bid	WB	ab10640, abcam	1:500	4℃, Overnight
ABCB1	WB	#12683, Cell Signaling Technology	1:1000	4℃, Overnight
ABCG2	WB	E-AB-30393, Elabscience	1:500	4℃, Overnight
ABCC1	WB	#14685, Cell Signaling Technology	1:500	4℃, Overnight
GST	WB	#13-6700, Thermo Fisher	1:2000	4℃, Overnight
ABCB1	IHC	#12683, Cell Signaling Technology	1:400	4℃, Overnight
PI3K p100α	WB	#4249, Cell signaling	1:500	4℃, Overnight
PI3K p100β	WB	#3011, Cell signaling	1:500	4℃, Overnight
phospho-mTOR	WB	#2976, Cell Signaling	1:500	4℃, Overnight
mTOR	WB	#2983, Cell Signaling	1:500	4℃, Overnight
phospho-AKT	WB	#4060, Cell Signaling	1:500	4℃, Overnight

AKT	WB	#9272, Cell Signaling	1:500	4℃, Overnight
Ki67	WB	ab92742, abcam	1:2000	4℃, Overnight
PCNA	WB	GTX100539, Genetex	1:400	4℃, Overnight
CDK1	WB	#9116, Cell signaling	1:2000	4℃, Overnight
CDK2	WB	#2546, Cell signaling	1:2000	4℃, Overnight
CDK4	WB	#12790, Cell signaling	1:1000	4℃, Overnight
CDK6	WB	#13331, Cell signaling	1:2000	4℃, Overnight
Cyclin E1	WB	#4129, cell signaling	1:1000	4℃, Overnight
Cyclin B1	WB	#4138, cell signaling	1:1000	4℃, Overnight
ABCB1	WB	#12683, Cell Signaling Technology	1:1000	4℃, Overnight

Abbreviations:
WB, Western blot; IP: Immunoprecipitation; IHC: Immunohistochemistry

Supplementary Table S2. Sequence of the oligonucleotides for Quantitative real-time PCR assays

Target		5' to 3'
18s rRNA	Forward	**GTAACCCGTTGAACCCCATT**
	Reverse	**CCATCCAATCGGTAGTAGCG**
ABCB1 mRNA	Forward	**GCTCCTGACTATGCCAAAGC**
	Reverse	**TCTTCACCTCCAGGCTCAGT**

Supplementary Table S3. List of plasmid constructs used.

Plasmid	Vector
Flag-USP21	pFlag-CMV2
Flag-USP12	pFlag-CMV6
Flag-USP7	pFlag-CMV2
Flag-USP7 1-500	pFlag-CMV2
Flag-USP7 500-1100	pFlag-CMV2
GST-USP7 1-209	pGEX4T1
GST-USP7 210-500	pGEX4T1
